# Ethyl Acetate Fraction and Isolated Phenolics Derivatives from *Mandevilla moricandiana* Identified by UHPLC-DAD-ESI-MS^n^ with Pharmacological Potential for the Improvement of Obesity-Induced Endothelial Dysfunction

**DOI:** 10.3390/pharmaceutics13081173

**Published:** 2021-07-29

**Authors:** Leticia L. D. M. Ferreira, Valéria de F. Leão, Cinthya M. de Melo, Thelma de B. Machado, Ana Claudia F. Amaral, Leandro L. da Silva, Naomi K. Simas, Michelle F. Muzitano, Ivana C. R. Leal, Juliana M. Raimundo

**Affiliations:** 1 Pharmacology of Bioactive Products Research Group, Federal University of Rio de Janeiro—Macaé Campus, Macaé 27930-560, RJ, Brazil; lldmf@ufrj.br (L.L.D.M.F.); valerialeao@ufrj.br (V.d.F.L.); maciel.cinthya@ufrj.br (C.M.d.M.); lelouback@macae.ufrj.br (L.L.d.S.); 2 Laboratory of Natural Products and Biological Assays, Pharmacy Faculty, Health Sciences Center, Federal University of Rio de Janeiro, Rio de Janeiro 21941-902, RJ, Brazil; naomi@pharma.ufrj.br; 3 LAQV-REQUIMTE, Department of Chemical Sciences, Faculty of Pharmacy, University of Porto, 4050-313 Porto, Portugal; thelma_machado@id.uff.br; 4 Faculty of Pharmacy, Federal Fluminense University, Niterói 24241-000, RJ, Brazil; 5 Laboratory of Medicinal Plants and Derivatives, Farmanguinhos, Oswaldo Cruz Foundation, Rio de Janeiro 21041-250, RJ, Brazil; ana.amaral@fiocruz.br; 6 Laboratory of Bioactive Products, Federal University of Rio de Janeiro—Macaé Campus, Macaé 27933-378, RJ, Brazil; mfmuzitano@macae.ufrj.br

**Keywords:** *Mandevilla moricandiana*, polyphenols, flavonols, procyanidin, triterpenes, vasodilation, antioxidant, endothelial dysfunction, obesity

## Abstract

Endothelial dysfunction in obesity plays a key role in the development of cardiovascular diseases, and it is characterized by increased vascular tonus and oxidative stress. Thus, this study aimed to investigate the vasodilatory and antioxidant activities of *Mandevilla moricandiana* ethyl acetate fraction and subfractions. Vascular effects were investigated on aorta isolated from control and monosodium glutamate (MSG) induced-obese Wistar rats, and antioxidant activity was assessed by 2,2-diphenyl-1-picrylhydrazyl (DPPH) and oxygen radical absorbance capacity (ORAC) methods. The ethyl acetate fraction (MMEAF) induced a concentration-dependent vasodilation on aortic rings through the NO pathway, with the involvement of histamine H1 and estrogen ERα receptors and showed potent antioxidant activity. In aorta of MSG obese rats, maximal relaxation to acetylcholine was increased in the presence of MMEAF (3 µg/mL), indicating that MMEAF ameliorated obesity-induced endothelial dysfunction. Quercetin and kaempferol aglycones and their correspondent glycosides, as well as caffeoylquinic acid derivatives, A-type procyanidin trimer, ursolic and oleanolic triterpenoid acids were identified in subfractions from MMEAF and seem to be the metabolites responsible for the vascular and antioxidant activities of this fraction.

## 1. Introduction

Cardiovascular diseases (CVD) are the leading cause of death worldwide, being responsible for 17.9 million deaths in 2019 [1]. Endothelium dysfunction is involved in both the development and progression of CVD and is also present in risk factors for these diseases. It plays a key role in structural and functional vascular changes observed in atherosclerosis [2,3], coronary artery disease [4], hypertension [5,6], and obesity [7,8]. The increasing incidence of obesity represents a global health problem since it is commonly associated with hypertension, type 2 diabetes, and atherosclerosis, besides being considered an independent risk factor for CVD [9].

Endothelium dysfunction is characterized by an imbalance between endothelial vasodilator and vasoconstrictors factors, reduced nitric oxide (NO) production and bioavailability, increased vascular tone, impaired coagulation, and an ambient of inflammation and oxidative stress [10,11]. The imbalance between antioxidants and oxidants results in endothelial NO synthase (eNOS) uncoupling, reduced NO bioavailability, lipid peroxidation, and the activation of mechanisms of vascular inflammation, which lead to vascular dysfunction and remodeling [12,13]. Obesity has been associated with a chronic inflammatory state, where adipose tissue produces pro-inflammatory mediators, such as tumor necrosis factor-alpha [7]. This cytokine contributes to endothelial dysfunction through the inhibition of eNOS activity and the induction of reactive oxygen species production [14,15]. In addition, endothelial pro-inflammatory activation leads to upregulation of adhesion molecules, the release of chemokines and cytokines, infiltration of leukocytes, and establishment of a prothrombotic state. These events are closely related to the development and progression of CVD, such as atherosclerosis [16,17,18]. Natural polyphenols, which include flavonoids and non-flavonoids molecules, are secondary plant metabolites with multiple biological effects [19]. The consumption of polyphenols-rich food has been associated with a reduction in cardiovascular risk [20,21,22], and phenolic compounds have shown therapeutic potential for the treatment of cardiovascular diseases [23,24]. A number of polyphenols induce endothelium-dependent vasodilation [25] and can protect against and/or attenuate endothelial dysfunction through multiple mechanisms, such as reducing oxidative stress and vascular inflammation [26].

The genus *Mandevilla* (Apocynaceae) includes 71 species widely distributed throughout Brazil [27]. *Mandevilla illustris* and *M. velutina* are the most studied species, especially regarding their anti-inflammatory, anti-snake venom, and bradykinin antagonist activities [28,29,30]. *M. moricandiana* is an endemic species mainly found in the southeast and northeast of Brazil and occurs at the Restinga de Jurubatiba National Park (RJ). Among our efforts to study the plant biodiversity of this park, we have previously shown that the hydroalcoholic extract of leaves of *M. moricandiana* induces NO-dependent vasodilation, partially through the activation of histamine (H1) and estrogen (ERα) receptors. This effect seemed to be mediated by flavonoids that are present in the extract [31]. Another aspect that encourages further chemical and pharmacological studies about *M. moricandiana* is that micropropagation and in vitro conservation techniques have been previously described [32,33] and could support the sustainable management of the species.

Therefore, the aim of this work was to evaluate the vasodilatory and antioxidant effects of the ethyl acetate fraction, subfractions, and isolated compounds of the hydroalcoholic extract of leaves from *M. moricandiana*. In addition, the effect of the phenolics-derivatives enriched ethyl acetate fraction on vascular reactivity of aorta isolated from monosodium glutamate (MSG)-induced obese rats was investigated in order to evaluate its pharmacological potential for improving endothelial dysfunction.

## 2. Materials and Methods

### 2.1. Reagents

Phenylephrine, acetylcholine, N^G^-nitro-L-arginine methyl ester (L-NAME; ≥98%), diphenhydramine (≥98%), fulvestrant (≥98%), 2,2-diphenyl-1-picrylhydrazyl (DPPH), fluorescein, 2,2′-azobis (2-methylpropionamidine) dihydrochloride (AAPH), and Trolox ((±)-6-hydroxy-2,5,7,8-tetramethylchromane-2-carboxylic acid) were purchased from Sigma-Aldrich^®^ (St. Louis, MO, USA). Methanol, acetonitrile, formic acid, and ethyl acetate were purchased from Tedia^®^ (Fairfield, CT, USA).

### 2.2. Plant Material and Extraction

The leaves of Mandevilla moricandiana were collected at the Restinga de Jurubatiba National Park (22°27′ S, 41°65′ W), Quissamã, Rio de Janeiro, Brazil, in March 2015. The botanical identification of the vegetal species was performed by Tatiana Ungaretti Paleo Konno and a voucher specimen has been deposited at the Herbarium of the Federal University of Rio de Janeiro, under the number RFA38748. Leaves (99 g) were air-dried at 40 °C, and then exhaustively extracted with ethanol/H_2_O (7:3) by static maceration at room temperature. The hydroalcoholic crude extract of the leaves from M. moricandiana (MM) was obtained after the removal of the solvent under reduced pressure by a rotatory evaporator. The MM extract was solubilized in 350 mL of MeOH/distilled water (9:1) under agitation and was submitted to successive liquid–liquid partitions with organic solvents in the following eluotropic order: n-hexane (MMHF), dichloromethane (MMDCMF), ethyl acetate (MMEAF), and n-butanol (MMBF). After partition with n-butanol, the remaining aqueous fraction (MMAF) was obtained, and it was submitted to freezing followed by lyophilization (Freeze Dryer: L101 Liotop, São Paulo, Brazil), as previously described by our group [31].

### 2.3. Purification of MMEtAc Fraction by Glass Column Chromatography

MMEAF (1.93 g) was re-suspended in methanol (100 μL) and purified by a Sephedex (LH-20) (Fluka^®^) column chromatography (h = 54 cm, d = 2.1 cm, filled with stationary phase = 36 cm). The following solvent systems were used as eluent, in this order: 2 L of methanol (Tedia^®^), 1 L of methanol: water (1:1), 500 mL of water, and 2 L of methanol. The solvent flow rate used for the chromatographic separation was set at 2 mL/min. Ten subfractions were afforded (MMEAF—A, 600mL; MMEAF—B, 400mL; MMEAF—C, 300 mL; MMEAF—D, 100 mL; MMEAF—E, 100 mL; MMEAF—F, 500 mL; and MMEAF—G, 950 mL).

### 2.4. Ultra-High Performance Liquid Chromatography Coupled to Diode Array Detector and Electrospray Ionization Mass Spectrometry (UHPLC-DAD-ESI-MS^n^)

The UHPLC-DAD-ESI-MS^n^ analysis was performed on a Thermo LCQ FLEET system (Thermo Fisher Scientific, Waltham, CA, USA) consisting of a FINNIGAN Surveyor MS Pump Plus, a FINNIGAN Surveyor Autosampler Plus, a dual detection system of a photodiode array detector (PDA), and a LCQ FLEET ion trap mass spectrometer equipped with an electrospray ionization (ESI) source (LCQ, Thermo Fisher Scientific, Waltham, CA, USA). Thermo Xcalibur version 2.2 software (Waltham, USA, 2011) was used for data acquisition and processing. The chromatographic condition established was: RP18 end capped column (2 μm) (Hibar HR 100 × 2.1 mm Purospher Star^®^); sample injection volume, 20 μL; the temperature of column oven, 25 °C; flow rate, 0.2 mL/min. The mobile phases adopted were formic acid 0.1% (A) and acetonitrile + formic acid 0.1% (B). A gradient elution protocol was used as follows: 0–10 min, 5–15% B; 10–20 min, 15–25% B; 20–35 min, 25–50% B; 35–50 min, 50–100% B; 50–55 min, 100% B; 55–60 min, 100–5% B; 60–65 min, 5% B. The PDA acquisition wavelength was set in the range of 190 to 600 nm. The samples were prepared through the dilution of 2 mg in 1000 μL methanol (2 mg/mL), and the chromatography runs were monitored at 190, 210, 254, and 365 nm wavelengths. The quantitative analyses were based on the peak relative area percentage. The mass spectrometer equipped with an electrospray ionization (ESI) source was operated in the negative ionization mode (ESI-(-)) with the final conditions as follows: electrospray voltage 4.5 kV and 5 kV, capillary temperature 350 °C, capillary voltage ± 24 V. Nitrogen was used as sheath and auxiliary gas. Full scan data acquisition was performed from *m*/*z* 120 to 1500 in negative MS scan mode. MS/MS experiments were performed by the collision of the precursor ions with helium gas. The collision energy value was set at 30% of the instrument maximum.

### 2.5. Purification by Semi-Preparative High-Performance Liquid Chromatography Coupled to Diode Array Detector (HPLC-DAD)

In order to isolate the substances present in MMEAF-B and MMEAF-D, the fractions were purified by the Shimadzu LC-20AT HPLC equipped with a DAD detector SPD M20A (Shimadzu^®^, Kyoto, Japan), using a semi-preparative column (LUNA C18; 250 mm × 10 mm × 5 µm). In this procedure, 60 mg of the sample MMEAF-B or 20 mg of sample MMEAF-D were applied, separated into three injections of 20 mg/200 μL for each sample. The ultraviolet (UV) detection was monitored at 254 nm. The components were separated using a gradient elution protocol consisting of formic acid solution (0.1%) in ultrapure water (A) and acetonitrile (B), such as the following: 0–10 min (5–15% B); 10–20 min (15–25% B); 20–35 min (25–50% B); 35–50 min (50–100% B); 50–55 min (100% B). The flow rate was set at 4 mL/min.

### 2.6. Nuclear Magnetic Resonance (NMR)

NMR spectra of the isolated substances were recorded on a VARIAN INOVA 500 MHz spectrometer (Varian Inc., Palo Alto, CA, USA) working at 499.78 (^1^H) and 125.68 MHz (^13^C) at the Institute of Natural Product Research (IPPN)—UFRJ/RJ; on a 400 MHz BRUKER Model: Avance II HD spectrometer equipped with a Bruker Prodigy BB0400S1 cryosonday or on a 400 MHz spectrometer at the Analytical Methodology Platform (PMA)—Farmanguinhos—Fiocruz/RJ. Bidimensional spectra (HSQC, HMBC, COSY) also proceeded. Chemical shifts (δ) were endorsed with tetramethylsilane (TMS) as internal standards (δ = 0, 1H) being expressed in ppm units and coupling constants (J) in Hertz (Hz). The pulse sequences used are all standard in the VNMRJ software, and the experiments were conducted at 25 °C. The samples were dissolved in deuterated methanol (CD_3_OD).

### 2.7. Animals

All experimental protocols were approved by the Animal Care and Use Committee at the Federal University of Rio de Janeiro under the license MAC019. Male Wistar rats were maintained on a 12 h light/dark cycle with controlled temperature (23 ± 2 °C) and free access to food and water.

### 2.8. Preparation of Aortic Rings for Isometric Tension Recording

Thoracic aortas were dissected from male Wistar rats (200–250 g). After the removal of connective tissue, the aorta was cut into 2–3 mm rings, which were suspended in organ baths filled with Krebs–Henseleit solution (118 mM NaCl; 4.7 mM KCl; 1.2 mM KH_2_PO_4_; 1.2 mM MgSO_4_; 2.5 mM CaCl_2_; 25 mM NaHCO_3_, and 11 mM glucose; pH 7.4; 37 °C) continuously gassed with carborgen gas (95% O_2_, 5% CO_2_). Aortic rings were mounted between two hooks, in which one was attached to a force transducer (MLT0201; AD Instruments, Bella Vista, New South Wales, Australia). Signals were digitalized (Power Lab 4/30; AD Instruments, Bella Vista, New South Wales, Australia) and stored on a computer for analysis using the software LabChart Pro (AD Instruments, Bella Vista, New South Wales, Australia). After an equilibrium period of 90 min under 1 *g* resting tension, aortic rings were contracted with phenylephrine (Phe; 10 µM), and the presence of functional endothelium was confirmed by a relaxation response to acetylcholine (ACh; 10 µM) greater than 80%. In some rings, the endothelium was mechanically removed, which was confirmed by the lack of relaxation in response to ACh [31].

### 2.9. Vasodilatador Effect

Concentration–response curves for MMEAF, subfractions, and isolated compound (0.1–30 μg/mL) were obtained in aortic rings with endothelium precontracted with 10 µM Phe. In order to determine the mechanisms involved in MMEAF-induced vasodilation, concentration–response curves for MMEAF were obtained in aortic rings without endothelium, or in aortic rings with endothelium pretreated (15 min) with the NO synthase inhibitor L-NAME (100 μM), the histamine H1 receptor antagonist diphenhydramine (10 μM), or the nonselective estrogen receptor antagonist fulvestrant (10 μM). The relaxation response was expressed as the percentage of maximal contraction induced by Phe, and the concentration necessary to induce 50% of maximal response (EC_50_) was calculated by non-linear regression analysis.

### 2.10. Effects of MMEAF on Vascular Reactivity of Aorta from Obese Rats

Obesity was induced in male Wistar rats through subcutaneous injections of MSG (4 g/kg body weight: MSG group) during the first 5 days of life. Control (CTL) group received hyperosmotic saline solution (1.25 g/kg body weight). Body weight and food intake were determined once a week. After euthanasia, perigonadal and retroperitoneal fat pads were collected and weighed. Obesity was confirmed by the increase in fat stores, despite the reduction in body weight, as described in our previous publications where MSG-induced obesity was used [34,35]. All MSG rats used in this study presented these characteristics. The thoracic aorta was dissected and prepared for isometric tension recording, as previously described in Section 2.8. In order to evaluate vascular reactivity, concentration–response curves for Phe (10^−9^–10^−5^ M) and ACh (10^−9^–10^−5^ M) were obtained in the absence and presence of 3 μg/mL of MMEAF. EC_50_ was calculated by non-linear regression analysis and expressed as pEC_50_ (negative log EC_50_ in Molar).

### 2.11. 2,2-Diphenyl-1-picrylhydrazyl (DPPH) Assay

The DPPH scavenging activity of MMEAF and subfractions was measured according to [36], with minor modifications. In 96-well microtiter plates, 125 μL of each methanolic solution of MMEAF and the correspondent fractions (1–200 µg/mL) was mixed with 50 μL of 300 µM DPPH methanolic solution and kept in the dark for 30 min at room temperature. Then, absorbances were measured at 517 nm using a spectrophotometer (UVM 340 spectrophotometer, Biochrom ASYS, Cambridge, UK). The blank consisted of 125 μL of samples and 50 μL of methanol, and negative control was 50 μL of DPPH and 125 μL of methanol. The standardized leaf extract of *Ginkgo biloba* (EGb761^®^; 1–200 µg/mL) and Trolox (1–200 µg/mL) were used as positive controls. Three independent experiments were performed in triplicate for each sample, and final results were expressed as a percentage of antioxidant activity (AA%). EC_50_ was calculated by non-linear regression.

### 2.12. Oxygen Radical Absorbance Capacity (ORAC) Assay

The ORAC of MMEAF and subfractions was measured according to [37], with minor modifications. To 20 µL of MMEAF or subfractions (0.25–2 µg/mL) was added 120 µL of 116.7 µM fluorescein solution in 96-well black plates. After a 10 min-period of incubation at 37 °C, 60 µL of 40 mM AAPH solution was added, and fluorescence was recorded at 480/530 nm, for 95 min at 10 min intervals by using a fluorimeter (Fluostar Optima; BMG Labtechnologies GmbH, Offenburg, Germany). Trolox (0.25–2 µg/mL) was used as positive control. The blank consisted of 80 μL of phosphate-buffered saline (PBS) and 120 μL of fluorescein, and negative control consisted of 20 μL of PBS, 60 μL of AAPH, and 120 μL of fluorescein. Three independent measures in triplicate were performed for each sample. Final results were expressed as mmol of Trolox equivalent (TE)/g of the sample by using the standard curves.

### 2.13. Statistical Analysis

Data are expressed as means ± S.E.M. Analyses were performed using Prism 5.0 software (GraphPad Software, La Jolla, CA, USA). Data were analyzed using a one-way analysis of variance followed by Tukey’s post-hoc test. Differences between groups were considered statistically significant when *p* < 0.05.

## 3. Results and Discussion

### 3.1. Chemical Analysis of MMEAF and Its Subfractions

**Figure 1:**
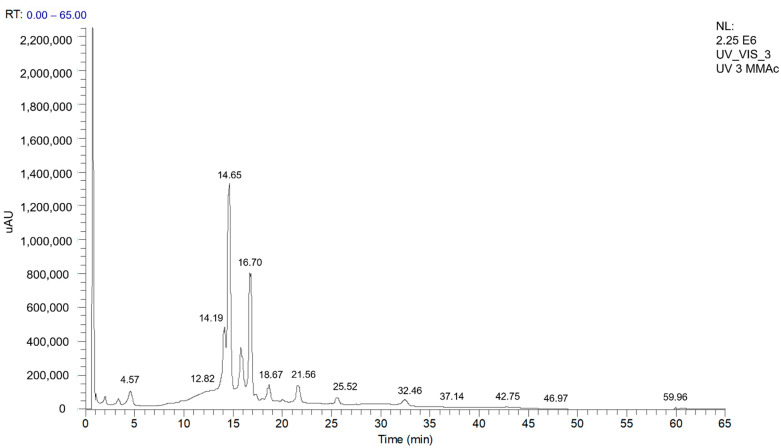
UHPLC-DAD-ESI-MS/MS chromatogram profile for MMEAF monitored at 254 nm.

MMEAF was analyzed by UHPLC-DAD-ESI-MS^n^, and the chromatogram wavelength monitored was set at 254 nm, as it is shown in Figure 1. Two constituents were observed as major compounds, such as that at 14.65 min (λ 258 and 352 nm) (41.76%) and at 16.70 min (λ 266 and 347 nm) (27.02%), with UV spectra characteristic of the benzyl (ring A) and cinnamoyl (ring B; C2-C3) portions, respectively, from flavonoid chemical structures. Minority compounds were also observed in 4.57 and 14.19–25.52 min, also with phenolic absorption characteristics, which will be discussed further in detail.

**Figure 2:**
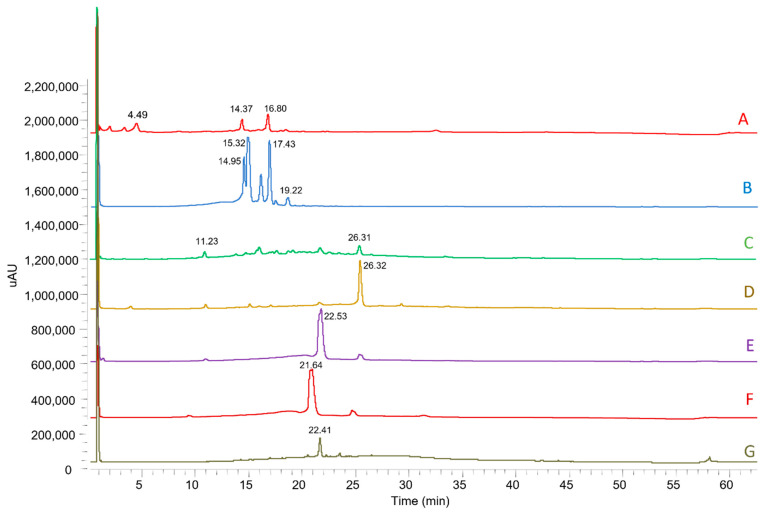
Chromatograms of the subfractions MMEAF-A–G analyzed by UHPLC-DAD-ESI-MS^n^ and monitored at 254 nm.

**Figure 3:**
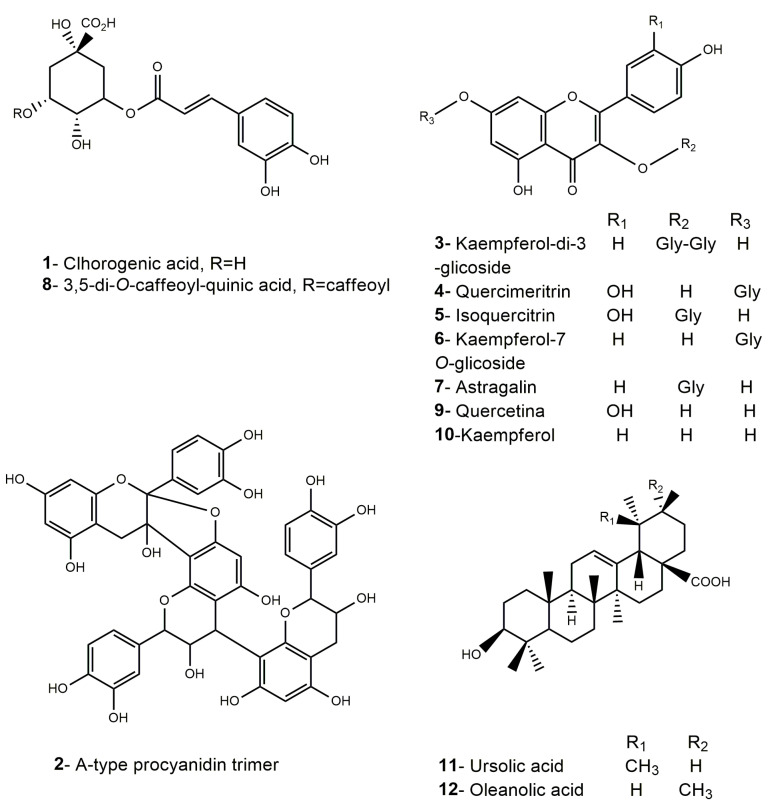
Chemical structures (1–12) of the compounds identified in MMEAF subfractions.

MMEAF was then purified by Sephadex column chromatography affording 7 subfractions (MMEAF-A-G) with 35.48; 36.86; 3.67; 2.16; 8.33; 1.76, and 2.00% yield, respectively, that were analyzed by UHPLC-DAD-MS/MS (Figure 2). UV spectra and MS data of each main peak allowed the identification of flavonols (aglycones and glycosides), caffeoylquinic acid derivatives, procyanidin, and pentacyclic-type triterpenes, as shown in Figure 3 and Table 1.

From MMEAF-A, UHPLC-DAD-ESI-MS/MS detected a compound (Rt = 4.49 min) with UV max absorption at 353 nm, which is typical of hydroxycinnamic acid derivatives [38]. It exhibited a pseudo molecular ion at *m*/*z* 353 [M-H]^−^ with a MS/MS fragmentation at *m*/*z* 191 [M-1-162]^−^ [M-H-sugar]^−^ suggestive of quinic acid, due to caffeoyl cleavage and sugar loss, a pattern that is compatible with the polyphenol chlorogenic acid (**1**). Compounds with pseudo molecular ions were also identified at *m*/*z* 609 [M-H]^−^ with MS2 fragmentation at *m*/*z* 284, suggested to be kaempferol-*di*-3-glycoside (**3**) (Rt = 14.37 min) [39], and, at *m*/*z* 447 [M-H]^−^, compatible with kaempferol-7-*O*-glucoside (**6**) (Rt = 16.60 min), which will be discussed further.

From MMEAF-B, UHPLC-DAD-ESI-MS/MS detected compounds suggestive of flavonols, including the peaks at Rt = 14.94 min and 15.33 min, both with deprotonated ions [M-H]^−^ at *m*/*z* 463, with MS/MS fragmentation at *m*/*z* 301 [M-1-162]^−^ [M-H-sugar]^−^ suggestive of glycosil moiety loss, compatible with two isomers of quercetin-*O*-hexoside, suggestively identified as quercimeritrin (**4**) and isoquercitrin (**5**), respectively. Both peaks at Rt = 16.60 min and 17.48 min corresponded to the pseudo molecular ions with *m*/*z* 447 [M-H]^−^ that presented two high abundance fragments at *m*/*z* 285 [M–H–162]^−^ and 284 [M–H–162]^−•^ indicating the loss of a hexose unit, being identified as kaempferol-7-*O*-glucoside (**6**) and astragalin (kaempferol-3-*O*-glucoside) (**7**), respectively. The glycosylation site of 3-OH in astragalin (**7**) was indubitably proved by NMR spectroscopic data, as will be discussed. In addition to these, it was also identified a peak at Rt = 19.22 min, with a deprotonated molecular ion [M-H]^−^ at *m*/*z* 515. As known, both *di*-caffeoyl-quinic acid (diCQA) and caffeoyl quinic acid (CQA)-glycosides produce an isobaric pseudo molecular ion at *m*/*z* 515. Unlike the diCQA, the CQA glycosides produce distinctive ions at *m*/*z* 341 ([caffeoyl glucoside-H]^−^) or/and 323 ([caffeoyl glucoside-H-H_2_O]^−^), that were not observed. An ion with MS/MS fragmentation at *m*/*z* 353 [M-1-162]^−^ was detected, produced by the loss of one of the caffeoyl moieties [M-H-caffeoyl]^−^, followed by subsequent fragmentation of this ion to furnish *m*/*z* 317 [M-H-caffeoyl- 2 H_2_O]^−^, *m*/*z* 299 [M-H-caffeoyl- 3 H_2_O]^−^ and *m*/*z* 173, indicating a diCQA. The weak signal of the ion at *m*/*z* 173 could indicate the absence of a C4 substituent, being tentatively assigned as 3,5-*O*-di-caffeoylquinic acid based on its fragmentation pattern and abundances (**8**) [40].

**Figure 4:**
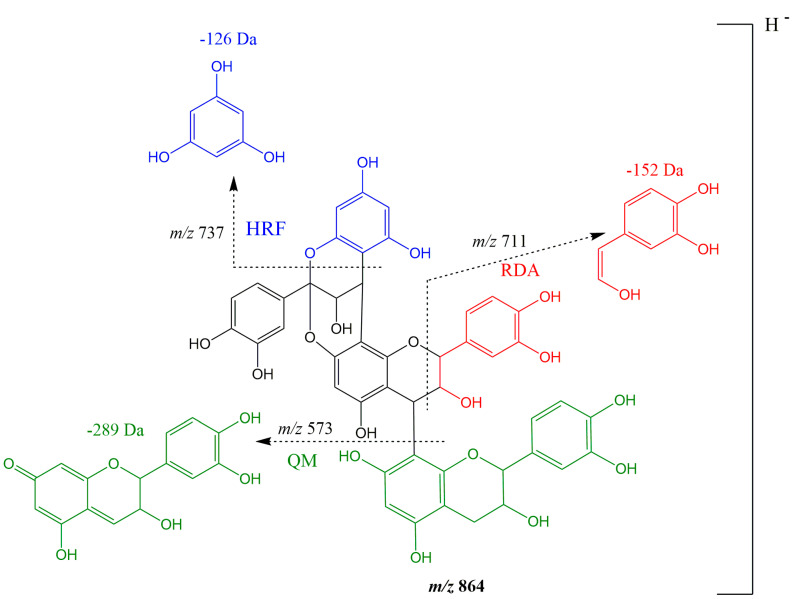
Fragmentation mechanisms proposed for the deprotonated molecule of A-type procyanidin trimer (ATT) by ESI(-)-MS/MS. In accordance with [41,42], we propose the trimer to be of type PC ATT [(E)C→A→(E)C→B→(E)C], in which abbreviations-related identifications of molecular species are described as follow: epicatechin (E), catechin (C), heterocyclic ring fusion (HRF), retro-Diels–Alder (RDA), quinone methide (QM). A-type linkage (A), which can be (C4 → C8), (C2 → O7) or (C4 → C6), (C2 → O7), B-type linkage (B), which can be (C4 → C8) or (C4 → C6). The C4 → C6 was rarely found to occur. The abbreviation E(C) indicates that the monomeric unit is indistinguishable using mass spectrometry.

MMEAF-D was subjected to a HPLC-DAD semi-preparative method, as described in the Material and Methods Section, affording an isolated compound with the deprotonated molecular ion *m*/*z* 447 [M-H]^−^ detected by UHPLC-DAD-ESI-MS/MS, suggesting the presence of kaempferol (**10**) (Rt = 26.32 min). From MMEAF-D, a compound with λmax at 280 nm (Rt = 11.21 min) and a deprotonated molecular ion *m*/*z* 863 [M-H]^−^ with four main fragment ions at *m*/*z* 711, *m*/*z* 573, *m*/*z* 451, and *m*/*z* 289 was identified. This ion was tentatively identified, based on previous data found in the literature, as a procyanidin (PC) (*epi*)-catechin trimer with a type-A linkage, thus being assigned as an A-type (*epi*)-catechin trimer (**2**) (5.5 mg; >95% purity at 254 nm), the same as proposed by [41] by LC-MS/MS analyses. Differently from others, the A-type trimer (ATT) has an extra bond between favan-3-ol units, which links the C-2 of the upper unit to the C-7 of the lower unit. The compound identified in MMEAF-D is likely a PC ATT with 1 A-bond (Figure 4). In general, the dissociation manner of PC polymers is similar to the dimers, but, in the case of ATT, it has two types of fragmentation patterns. For PC ATT, the quinone methide (QM) fission cleavage ions are present at *m*/*z* 289 ([M_B_-H (290-H)]^−^, QM fission at the terminal unit) and *m*/*z* 573 [M-290-H)]^−^, that produces the fragment *m*/*z* 451 [M-290-123((C_6_H_6_O_3_)-3H)]^−^ by heterocyclic ring fusion (HRF). Under this circumstance, the fragment ion at *m*/*z* 737 is also formed by HRF of *m*/*z* 863 [M-H]^−^ with the loss of 126 Da [M-H-126(C_6_H_6_O_3_)]^−^, while *m*/*z* 711 is formed by retro-Diels–Alder fission (RDAF) of the middle unit, with the loss of 152 Da [M-H-152]^−^ [42].

From MMEAF-C, the same substances as observed in MMEAF-D were detected but in different amounts concerning the percentage of relative area. Kaempferol (**10**) was detected as the major compound in MMEAF-D.

From MMEAF-F, quercetin (**9**) (Rt = 21.64 min) (90% relative area) was solely found, with UV max absorption at 267 and 370 nm.

The triterpenes ursolic acid (**11**), in the majority (55.76%), and oleanolic acid (8.11%) (**12**) were obtained in a mixture from MMEA-F-G and were identified by UHPLC-DAD-ESI-MS/MS by comparison with standards.

The fractions MMEA-F-B and MMEA-F-D were purified by semi-preparative HPLC, as described in the methods section, and the isolated compounds were structurally elucidated by ^1^H-NMR, ^13^C-NMR, HSQC, and HMBC spectroscopic data, as it is described below.

### 3.2. NMR Data

Quercetin (9) (37,4 mg): Rt = 21.64 min (C_15_H_10_O_7_), ^1^H NMR (400 MHz, CD_3_OD) δ (ppm):6.40 (1H, *d*, *J* = 2.0 Hz, H-8), 6.19 (1H, *d*, *J* = 2.0 Hz, H-6), 6.89 (1H, *d*, *J* = 8.5 Hz, H-5′), 7.64 (1H, *dd*, *J* = 8.5, 2.1 Hz, H-6′), 7.74 (1H, *d*, *J* = 2.1 Hz, H-2′). ESI-MS *m*/*z* 301.14 [M-H]^−^.

Quercimeritrin (4) (20 mg, *in a mixture with isoquercitrin*): Rt = 14.94 min (C_21_H_20_O_12_); 1H NMR (400 MHz, CD3OD) δ (ppm): 3.14–3.74 (6H, *m*, H-2”, H-3”, H-4”, H-5”, Ha-6”, Hb-6”), 5.14 (1H, *d*, *J* = 7.8 Hz, H-1”), 6.37 (1H, *d*, *J* = 2.1 Hz, H-8), 6.15 (1H, *d*, *J* = 2.0 Hz, H-6), 6.84 (1H, *d*, *J* = 8.8 Hz, H-5′), 7.60 (1H, *dd*, *J* = 8.5, 2.2 Hz, H-6′), 7.71 (1H, *d*, J = 2.1 Hz, H-2′). ESI-MS *m*/*z* 463.29 [M-H]^−^.

Isoquercitrin (5) (20 mg, *in a mixture with quercimeritrin*): Rt = 15.33 min (C_21_H_20_O_12_); ^1^H NMR (400 MHz, CD_3_OD) δ (ppm): 5.25 (1H, *d*, *J* = 7.6 Hz, H-1”), 6.39 (1H, *d,* *J* = 2.1 Hz, H-8), 6.20 (1H, *d*, *J* = 2.1 Hz, H-6), 6.87 (1H, *d*, *J* = 8.5 Hz, H-5′), 7.58 (1H, *dd*, *J* = 8.5, 2.1 Hz, H-6′), 7.71 (1H, *d*, *J* = 2.1 Hz, H-2′), 3.44 (1H, *t*, *J* = 8.9 Hz, H-3”), 3.36 (1H, *J* = 9.2 Hz, H-4”), 3,14 (1H, *ddd*, *J* = 2.3, 5.3, 9.6 Hz, H-5”), 3.72 (1H, *dd*, *J* = 2.3, 11.9 Hz, Ha-6”), 3.58 (1H, *dd*, *J* = 5.5, 11.9 Hz, Hb-6”). ESI-MS *m*/*z* 463.33 [M-H]^−^.

Kaempferol (10) (6 mg): Rt = 21.64 min (C_15_H_10_O_6_), ^1^H NMR (400 MHz, CD_3_OD) δ (ppm): 6.40 (1H, *d*, *J* = 1.8 Hz, H-8), 6.18 (1H, *d*, *J* = 1.8 Hz, H-6), 6.91 (2H, *d*, *J* = 8.8 Hz, H-3′, H-5′), 8.09 (2H, *d*, *J* = 8.8, H-2′, H-6′). ESI-MS *m*/*z* 285.15 [M-H]^−^.

Astragalin (7) (9 mg): Rt = 26.32 min (C_21_H_20_O_11_); ^1^H NMR (400 MHz, CD_3_OD) δ (ppm): 5.22 (1H, *d*, *J* = 7.5 Hz, H-1”), 6.36 (1H, *d*, *J* = 2.1 Hz, H-8), 6.17 (1H, *d*, *J* = 2.1 Hz, H-6), 6.85 (2H, *d*, *J* = 9.0 Hz, H-3′, H-5′), 8.02 (2H, *d*, *J* = 9.0 Hz, H-2′, H-6′), 3.39 (2H, m, H-3”, H-4”), 3,16 (1H, *ddd*, *J* = 2.3, 5.5, 9.7 Hz, H-5”), 3.65 (1H, *dd*, *J* = 2.3, 11.9 Hz, Ha-6”), 3.49 (1H, *dd*, *J* = 5.5, 11.9 Hz, Hb-6”). ESI-MS *m*/*z* 447.20 [M-H]^−^.

### 3.3. Vasodilator Effect of MMEAF

**Figure 5:**
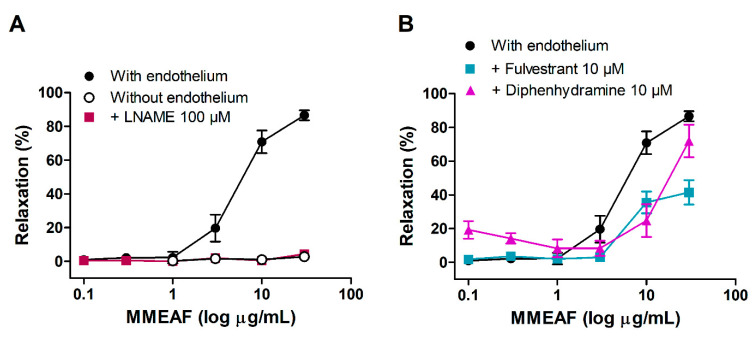
In (**A**), concentration–response curves for MMEAF in aorta with endothelium, in the absence and presence of L-NAME (100 µM), and in aorta without endothelium. In (**B**), concentration–response curves for MMEAF in the presence of fulvestrant (10 µM) or diphenhydramine (10 µM). Results are mean ± S.E.M of 5-7 experiments.

MMEAF induced a concentration-dependent relaxation of aortic rings with endothelium, with an EC_50_ of 6.91 ± 1.26 µg/mL. Maximal relaxation (30 µg/mL) was 86.56 ± 2.97%. Mechanical removal of endothelium completely inhibited the vasodilation induced by MMEAF, indicating that the effect depends on endothelium-derived factors (Figure 5A).

We have previously described that the hydroalcoholic extract of leaves of *M. moricandiana* induces endothelium-dependent vasodilation through the NO pathway, with the participation of histamine H1 and estrogen receptors [31]. Thus, the investigation of the mechanism of action of MMEAF was focused on this.

In the presence of L-NAME, the vasodilator effect of MMEAF was completely inhibited, indicating that the effect is dependent on NO release (Figure 5A). The involvement of histamine H1 and estrogen receptors in the activation of NO production was investigated through the pretreatment of aortic rings with diphenhydramine and fulvestrant, respectively. In the presence of diphenhydramine, the maximal effect of MMEAF was reduced to 51.33 ± 4.87% relaxation, and EC_50_ was increased to 26.41 ± 4.45 μg/mL (*p* < 0.05; Figure 5B). Pretreatment with fulvestrant did not alter maximal vasodilator response but induced a rightward displacement of the concentration–response curve of MMEAF, increasing EC_50_ to 13.94 ± 3.44 μg/mL (*p* < 0.05; Figure 5B).

These results indicate that the vasodilation induced by MMEAF is mediated, at least in part, through the activation of endothelial histamine H1 and estrogen receptors. Agonists of histamine H1 receptors lead to eNOS activation through the increase in intracellular calcium, while estrogen receptors signaling involves protein kinase B (Akt)-mediated Ser 1177 phosphorylation of eNOS [43].

Estrogen receptors are involved in the vascular effects of some polyphenols, such as resveratrol and delphinidin, found in red wine and black currant. These compounds induce the endothelial production of NO through the activation of estrogen ERα receptors [44,45,46]. Histamine H1 receptors have been implicated in the mechanism of vasodilation of the lignan eudesmin [47] and the extracts of *Cirsium japonicum* [48] and black currant [49].

### 3.4. Vasodilatory Activity of MMEAF Subfractions and Isolated Compound

**Figure 6:**
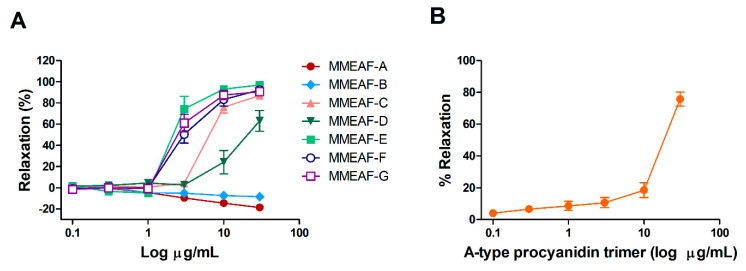
Concentration–response curves for subfractions (**A**) and A-type procyanidin trimer (**B**) in aortic rings with endothelium. Results are mean ± S.E.M of 4-7 experiments.

All subfractions obtained from the column chromatography of MMEAF were evaluated in aortas with endothelium. MMEAF-A and MMEAF-B showed no vasodilator effect at all concentrations tested. Instead, they produced a small increase in vascular tonus. Subfractions C-H induced intense vasodilation of aortic rings (Figure 6A). MMEAF-D was less effective and potent when compared to the other subfractions (Table 2).

The vasodilator effect of the A-type procyanidin trimer identified in subfractions C, D, and E was also evaluated. This compound induced a concentration-dependent relaxation of aorta with endothelium, with maximal relaxation of 75.77 ± 4.39% (Figure 6B; Table 2). As can be seen in Table 2, the isolated compound presented a greater maximal effect than MMEAF-D, but not statistically different from MMEAF-C and E. In contrast, the trimer was less potent than these two subfractions, suggesting that quercetin especially contributes to the higher potency of MMEAF-E, possibly through additive/synergistic actions with the trimer and other compounds.

As far as we know, this is the first report describing the vasodilator effect of an A-type procyanidin trimer. Procyanidins are polyphenols widely distributed in fruits, vegetables, nuts, and grains, where they are found as a mixture of oligomers [50]. Procyanidins have been shown to induce endothelium-dependent vasodilation, mainly via NO release [51,52], and the B-type procyanidin trimer C1 was shown to induce NO production in rat endothelial cells [53]. The biological effects of procyanidins vary according to the oligomeric, dimeric, and monomeric forms, highlighting the importance of the study of the different procyanidin compounds [50,53].

MMEAF-A, in which was identified chlorogenic acid, did not show vasodilator effect. Chlorogenic acid was shown to induce endothelium-dependent vasodilation of rat aorta, with the involvement of NO, prostacyclin, and endothelium-derived hyperpolarizing factor [54]. The lack of vasodilator effect of MMEAF-A may be related to the low concentration of this polyphenol in this subfraction.

MMEAF-B subfraction, which contains quercimeritrin, isoquercitrin, astragalin (kaempferol 3-*O*-glycoside), kaempferol 7-*O*-glycoside, and 3,5-dicaffeoylquinic acid, did not present vasodilatory activity, as was observed with MMEAF-A. Quercimeritrin [55,56] and isoquercitrin [57] have shown vasodilatory activity at high concentrations, which may explain the absence of effect of MMEAF-B. Furthermore, no reports of vasodilatory activity were found for the kaempferol glycosides identified in subfractions A and B.

Kaempferol was identified in subfractions C, D, and E, and quercetin was identified in subfractions E, F, and G. These flavonols are associated with cardiovascular protective effects due to many biological activities, including vasodilatory, antioxidant, and anti-inflammatory [58]. Quercetin has been shown to induce vasodilation in isolated vessels through endothelium-dependent [59] and -independent mechanisms [60], while the vasodilatory activity of kaempferol is partially dependent on endothelium [61,62]. Quercetin was the main compound found in subfraction F, which produced potent and intense relaxation of aortic rings with endothelium.

Oleanolic acid, which was identified in many subfractions of MMEAF with vasodilator effect, induces NO-dependent relaxation of rat superior mesenteric arteries [63] and aorta isolated from Wistar [64] and spontaneously hypertensive rats [65]. Similar effects are reported for ursolic acid in rat aorta [25,66].

### 3.5. Antioxidant Acitivity of MMEAF and Its Subfractions

Antioxidant activity of MMEAF and its subfractions was evaluated by using DPPH and ORAC assays, where *Ginkgo biloba* extract EGb761^®^ and quercetin were used as positive controls. All subfractions showed high antioxidant potential (Table 3).

Most compounds identified in MMEAF subfractions are reported as antioxidants, including chlorogenic acid, dicaffeoylquinic acid, quercetin, quercimeritrin, isoquercitrin, kaempferol, astragalin, and kaempferol 7-*O*-glycoside [58,67,68,69].

For the antioxidant activity, it is known that the presence of hydroxyl groups in the aromatic rings A and B are extremely important for the antioxidant activity [70]. However, in addition to the presence of the hydroxyl groups, their position also influences the antioxidant activity, which can be attributed to the presence of the hydroxyl groups at the C3, C3’, and C4’ positions [71]. In most plants, flavonoid glycosides occur at the C3 position, such as 3-*O*-glycosides [71]. The substitution of the hydroxyl group in C3 by sugars can reduces the antioxidant activity of flavonoids [72]. These data are in agreement with the results presented by the MMEAF-B fraction, which contains isoquercitrin and astragalin as major compounds, totaling 65.27% percentage of relative area, in addition to other glycosides, and presented lower antioxidant activity by the ORAC method (0.85 ± 0.05 mmol Eq Trolox/g sample). Among the fractions, by this same method, MMEAF-A presented the best activity, being statistically equal to the control quercetin. This fraction is also mainly composed of glycosides, such as kaempferol-di-3-glycoside and kaempferol-7-*O*-glycoside, but also by chlorogenic acid.

By the DPPH method, the most active fraction was MMEAF-F, which is mainly composed of quercetin (90%), which justifies the activity being quite similar (no statistical difference) to the commercial control quercetin, followed by MMEAF-E, composed mainly of kaempferol (29%). The aglycone quercetin has a catechol group in the B ring, that is, OH groups in 3,4 positions in the molecule of the phenolic compound. In this case, the hydroxyl groups in the ortho position could allow the formation of two hydrogen bonds with the two oxygen atoms of the peroxyl radical, and it led to a compact reactant complex, enhancing antioxidant activity. In addition to the presence of hydroxyls, another important characteristic for the antioxidant activity is the presence of the C2-C3 conjugated double bond with a carbonyl group in C4 [71]. It is important to highlight that all flavonoids identified were from the group of the flavonols, so they were constituted by the conjugated double bond in C2-C3, and this characteristic cannot be correlated to the different activity observed.

### 3.6. Effects of MMEAF on Vascular Reactivity of Aorta from Obese Rats

Since MMEAF presented both vasodilatory and antioxidant activities, we hypothesized that this dual effect could improve endothelial dysfunction. Thus, we evaluated the effects of MMEAF on vascular reactivity of aorta isolated from obese rats. Obesity was induced by early postnatal treatment with MSG, when the blood–brain barrier is not formed, which results in the development of hypothalamic obesity. This model presents common characteristics of clinical obesity, such as increased visceral adiposity, insulin resistance [73], systemic oxidative stress, reduced NO endothelial production, and endothelium dysfunction [74,75].

**Figure 7:**
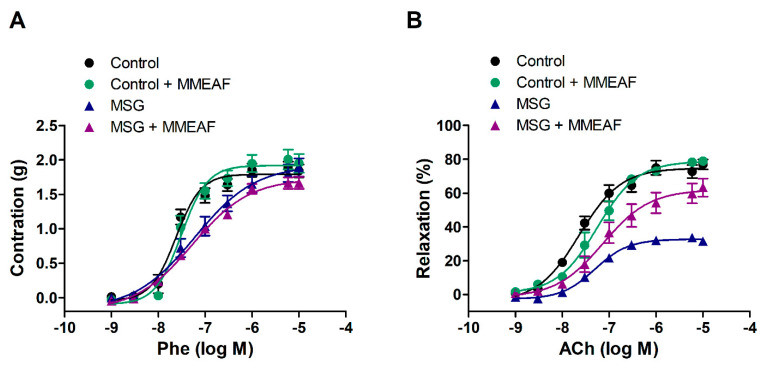
Effects of MMEAF on vascular reactivity to Phe (**A**) and ACh (**B**) in control and MSG aortic rings. Results are mean ± S.E.M of 5–6 experiments.

Aortic rings from MSG group showed reduced endothelium-dependent vasodilation to ACh compared to the control group, indicating the presence of endothelium dysfunction, as we have previously shown [34,35]. Maximal relaxation in response to ACh was 76.91 ± 2.94% and 31.60 ± 1.85% in control and MSG aorta, respectively (*p* < 0.05). Although maximal contraction induced by Phe was not altered, pEC_50_ was significantly altered in aorta from MSG rats (Figure 7; Table 4).

Incubation of aortic rings with 3 μg/mL MMEAF for 15 min did not change vascular reactivity of aorta from control rats or contractile response to Phe of aorta from MSG rats (Figure 7; Table 4). On the other hand, it significantly increased ACh-induced vasodilation in aorta from obese rats to 63.34 ± 5.36%, indicating that MMEAF improved obesity-induced endothelium dysfunction (Figure 7B; Table 4).

The effect of MMEAF was only observed in aorta from obese rats, where NO bioavailability was reduced. In an ambient of overproduction of reactive oxygen species, NO reacts with superoxide anion to form peroxynitrite, which oxides the eNOS cofactor tetrahydrobiopterin, leading to eNOS uncoupling and reduced NO bioavailability [13]. Thus, the improvement of ACh-induced vasodilation in aortic rings from obese rats may be related to the free radical scavenging activity of MMEAF. In addition, the activation of NO production in aorta could contribute to the effect of MMEAF. Considering the important role of the interaction between oxidative stress and inflammation in obesity-induced endothelial dysfunction, further studies are necessary to investigate if MMEAF could also reduce vascular inflammation.

The phytochemical analysis of MMEAF revealed the presence of different phenolic compounds that have protective vascular effects, which could additively and/or synergistically contribute to the effects of this fraction on aorta from obese rats. Studies have shown that polyphenolic mixtures present synergistic and additive antioxidant and vascular effects [76,77,78,79]. In this respect, quercetin and kaempferol synergistically acted in the reduction in H_2_O_2_-induced oxidative stress [80].

Kaempferol was shown to protect against paraquat-induced vascular injury by reducing oxidative stress and inflammation [81], and to reduce atherosclerotic formation and endothelial dysfunction in apolipoprotein E-deficient mice [82]. However, there are no reports regarding the effects of the kaempferol glycosides identified in MMEAF on endothelial dysfunction.

Quercetin and kaempferol presented anti-inflammatory activity in cytokine-stimulated human umbilical vein endothelial cells (HUVECs), where both flavonols inhibited the expression of adhesion molecules and cyclooxygenase-2 [83]. Quercetin reduced NO and anion superoxide imbalance induced by angiotensin II in HUVECs [84] and rat aorta [85], as well as restored endothelial function in spontaneously hypertensive rats [86] and in deoxycorticosterone acetate–salt hypertensive rats treated with this flavonol [87]. Pretreatment of aortic rings isolated from streptozotocin-induced diabetic rats with quercetin improved the relaxation response to ACh, which was mediated by its free radical scavenging activity [88]. The quercetin glycoside isoquercetrin was shown to inhibit high glucose-induced endothelial apoptosis in HUVECs [89].

Oleanolic acid improved endothelium-dependent vasodilation in high glucose treated-aorta, as well as restored NO production in HUVECs exposed to high glucose [90]. Ursolic acid treatment increased serum NO and prostacyclin levels and improved morphological parameters of aorta isolated from high choline-fed mice [91].

## 4. Conclusions

In this work, we investigated the phytochemical and pharmacological properties of MMEAF in order to evaluate its potential as a source of phenolic compounds with vascular protective actions. Our results showed that MMEAF is a polyphenol-enriched fraction, specially constituted by the flavonols kaempferol and quercetin and their glycosides, as well as phenylpropanoid derivatives, with potent antioxidant activity and NO-mediated vasodilator effect. Moreover, MMEAF restores endothelium-dependent vasodilation in aortas from MSG-induced obese rats, indicating its pharmacological potential for conditions associated with endothelium dysfunction. This effect seems to be mediated, at least in part, by the antioxidant effect of MMEAF and the consequent reduction in vascular oxidative stress. We also described, for the first time in this vegetal species, an A-type (epi)catechin trimer with vasodilatory activity, that should be investigated on deep concerning the effect observed.

## Figures and Tables

**Table 1:** Retention time (Rt), UV spectra, and MS/MS data of the compounds identified in the MMEAF subfractions (MMEAF-A-G).

Nº	Identification	Rt (min)	λmax (nm)	[M-H]^−^	MS2 Fragments (% Relative Area)	MMEAF-A	MMEAF-B	MMEAF-C	MMEAF-D	MMEAF-E	MMEAF-F	MMEAF-G
1	Chlorogenic acid	4.49	239,325	353	191 (100)	+						
2	Procyanidin A-type trimer	11.21–11.25	195, 280	863	711 (100), 411 (20), 451 (19), 573 (19)			+	+	+		
3	kaempferol-*di*-3-glycoside	14.37	258,351	609	-	+						
4	Quercimeritrin	14.94	259, 353	463	302 (100), 300 (64)		+					
5	Isoquecitrin	15.33	262, 347	463	302 (100), 300 (54)		+					
6	Kaempferol-7-*O*-glycoside	16.60–16.80	266, 347	447	284 (100), 285, (93), 255 (39)	+	+					
7	Astragalin (Kaempferol-3-*O*-glycoside)	17.48	264, 347	447	284 (100), 285, (60), 255 (31)		+					
8	3,5-dicaffeoylquinic acid	19.22	195, 216 e 329	515	353 (100), 299 (16), 255 (10), 203 (18), 173 (11)		+					
9	Quercetin	21.64–22.52	267, 370	301	273 (14), 179 (100), 151 (68)					+	+	+
10	Kaempferol	26.26–26.32	267, 366	285	239 (100), 229 (63), 241 (52), 242 (81), 257 (92)			+	+	+		
11	Ursolic acid	47.54–47.59	-									+
12	Oleanolic acid	47.98–48.91	-									+

+ represents the presence of the substance at 254 nm.

**Table 2:** Half maximal effective concentration (EC_50_) and maximal effect (E_max_) of MMEAF, subfractions and A-type procyanidin trimer for the vasodilator effect.

Sample	EC_50_ (μg/mL)	E_max_^1^ (% Relaxation)
MMEAF	5.56 ± 1.13 a	85.05 ± 3.64 a
MMEAF-A	ND ^2^	ND
MMEAF-B	ND	ND
MMEAF-C	7.37 ± 0.88 a	87.03 ± 3.30 a
MMEAF-D	22.43 ± 5.73 b	63.20 ± 9.74 b
MMEAF-E	2.77 ± 0.12 a	97.02 ± 0.79 a
MMEAF-F	4.12 ± 0.89 a	92.33 ± 2.80 a
MMEAF-G	3.33 ± 0.75 a	90.62 ± 3.86 a
A-type procyanidin trimer	18.22 ± 1.92 b	75.77 ± 4.39 a

^1^ At 30 μg/mL.^2^ Not determined. Results are mean ± S.E.M of 4-7 experiments. Different letters indicate statistically significant differences between samples (*p* < 0.05; One-way ANOVA followed by Tukey post hoc test).

**Table 3:** Antioxidant effect of MMEAF and subfractions assessed by DPPH and ORAC assays.

Sample	DPPH EC_50_ (μg/mL)	ORAC (mmol TE ^1^/g Sample)
MMEAF	8.57 ± 0.23 b	8.28 ± 0.25 a
MMEAF-A	17.89 ± 1.48 d	6.77 ± 0.03 a,d
MMEAF-B	6.19 ± 0.16 b	0.87 ± 0.02 c
MMEAF-C	6.30 ± 0.25 b,e	3.07 ± 0.19 b
MMEAF-D	11.47 ± 0.30 f	5.06 ± 0.13 b,d
MMEAF-E	3.38 ± 0.12 a	3.87 ± 0.25 b
MMEAF-F	2.08 ± 0.28 a	3.58 ± 0.02 b
MMEAF-G	5.36 ± 0.24 e	3.78 ± 0.89 b
EGB761^®^	26.69 ± 1.26 c	4.62 ± 0.22 b
Quercetin	2.70 ± 0.10 a	6.75 ± 0.76 a,d

^1^ Trolox equivalent. Results are mean ± S.E.M. of 3 independent experiments. Different letters indicate statistically significant differences between samples (*p* < 0.05; One-way ANOVA followed by Tukey post hoc test).

**Table 4:** Half maximal effective concentration (pEC_50_) and maximal effect (E_max_) of Phe and ACh.

Group	Phe		ACh	
	pEC_50_	Emax (g)	pEC_50_	Emax (%)
CTL	7.63 ± 0.07	1.84 ± 0.09	7.64 ± 0.05	76.91 ± 2.94
CTL + MMEAF	7.50 ± 0.07	1.95 ± 0.14	7.24 ± 0.14	78.74 ± 2.67
MSG	7.22 ± 0.13 *	1.89 ± 0.13	7.29 ± 0.03 *	31.60 ± 1.85 *
MSG + MMEAF	7.28 ± 0.62	1.67 ± 0.09	7.08 ± 0.12	63.34 ± 5.36

Results are mean ± S.E.M of 5-6 experiments. * *p* < 0.05 compared to CTL (One-way ANOVA followed by Tukey post hoc test).

## Data Availability

The data presented in this study are contained within the article.
